# Can countries shape the association between cumulative adversity and old-age health?

**DOI:** 10.3389/fpubh.2024.1364868

**Published:** 2024-05-15

**Authors:** Michal Levinsky

**Affiliations:** The Paul Baerwald School of Social Work and Social Welfare, Hebrew University of Jerusalem, Jerusalem, Israel

**Keywords:** lifetime cumulative adversity, Europe, self-rated health, SHARE, inequality theory

## Abstract

**Introduction:**

The present study examined the relationships of Lifetime Cumulative Adversity (LCA) and country inequalities, as well as the interactions between them, with the self-rated health (SRH) in old age.

**Methods:**

Using data from the Survey of Health, Aging and Retirement in Europe (SHARE), the study regressed self-rated health on Lifetime Cumulative Adversity and country-level inequality indices across European countries in two points in time. The analysis also considered adversity–inequality interactions, controlling for confounders. The sample was comprised of 28,789 adults, aged 50 to 80, from 25 European countries and Israel.

**Results:**

The findings pointed out that LCA is negatively associated with SRH, but democracy and welfare regimes modify the ill effects of LCA on health. These effects are reduced as the LCA level increases. The effects remained significant over two measurement time-points over three years, showing that life-course trajectories may be shaped by individual accumulated risk exposure to stress, along with inequalities at the society level.

**Discussion:**

The study provides constructive and important guidance for decreasing the harmful effect of lifetime adversity in old age, by the modification of the country’s welfare policies.

## Introduction

In recent decades, the percentage of the aging population in Europe has been increasing at an unprecedented rate, coupled with an increase in life expectancy. One of the major social challenges in this new trend is the rise of chronic health conditions in older age (1). The cumulative inequality theory (2) takes a lifespan perspective on the health deterioration throughout aging process, suggesting that individual and social levels shape inequality trajectories in life. That is, individual exposure to risk, along with social systems features (i.e., stratifying society according to class, race, and income), generates stress that leads to biological changes that accelerate the aging process and thus create health inequality.

Individual exposure to risk can be exemplified by Lifetime Cumulative Adversity (LCA), which refers to the accumulated exposure throughout life to a wide range of objective, external, stressful events fraught with traumatic reactions (3). LCA has been frequently investigated within the framework of cumulative inequality theory (4, 5), and is widely discussed as a negative predictor of mental and physical health in old age (6–8). Recent evidence suggests that LCA is related to the deterioration in self-rated health (SRH) over years during aging (4, 5, 9).

As mentioned, beyond individual exposure to risk, a person’s environment can be related to health, specifically in old age (10–12). Country of origin is one of the environmental factors differentiating many health circumstances (13, 14). Countries are complex entities and may differ by many indicators. Various economic and social mechanisms create the types of inequality present in a country, thereby associated with the health of the older adult living within it (13–16). The present study addressed three distinct dimensions of countries’ inequality generally used in the literature to explain health differences between countries: income inequality, democracy level, and welfare-state policies.

Income inequities may adversely impact population health because of their adverse effects on social capital: In societies with high economic disparities, the social capital eroded trust in others (17, 18). This lack of trust, indicating that people do not feel they can rely on others, exert constant psychosocial stress and is an important explanatory variable for the link between inequality and health (19). Political institutions may also alter the association between inequality and health. Some argue that living in a particular political system potentially alters the messages individuals receive about whether inequality is large or small, good or bad, and this messaging, in turn, might affect the beliefs about inequality and influence health (20). Following those different beliefs, studies found higher rates of perceived health in more democratic countries (20). Lastly, welfare states’ social benefits are considered essential for the health of the aging population (21, 22). Thus, for example, people in Scandinavian and Western welfare states, considered more social welfare-oriented, had better SRH scores than the Southern and Eastern European states (23). In general, the type of welfare state, by means of welfare regimes typology, accounted for approximately half of the national-level variation in health inequalities among European countries (23).

As described, it is well established that both individual adversities and environmental factors are related to inequality in the health of old age. However, little attention has been paid to the effect of country inequality on the individual adversities–health correlation. The interplay between the individual exposure to adversity and country inequality on old-age health has been investigated mainly with reference to early-life adversity and socioeconomic circumstances throughout life within welfare regimes. For example, a recent study found that in all welfare regimes early-life socioeconomic circumstances had a long-lasting association with perceived health, but that adult-life socioeconomic circumstances attenuated or strengthen this association differently across welfare regimes (24). Another study indicated that unfavorable childhood conditions exhibited a harmful influence on individuals’ chances for healthy aging across all European welfare states considered in the study (25), but that certain types of welfare regimes can strengthen the relationship between childhood adversity and frailty in old age (26). Nevertheless, those existing studies focused on the consequences of *childhood* adverse events on old-age health, whereas the effect of *lifetime* events on trajectories of old-age health has been studied only to a limited degree (24). Furthermore, although countries are complex entities and may differ by many aspects, to date there is not much evidence regarding the interplay between the various country inequality indicators and the *individual* cumulative adversity. In addition, most studies used a cross-sectional approach. The current study extends previous studies by investigating the interplay between LCA and various country indicators on SRH over a period of three years, with two measurement points in time.

Finally, research indicates that an individual’s health in later life is correlated to a person’s sociodemographic background. Thus, health considered in the present study has been found to be related to factors such as age, gender, living arrangements, and socioeconomic status (27–30). Consequently, these variables will be controlled.

Given the absence of literature regarding the interplay between individual exposure to cumulative life adversity and country inequality on old-age health, the present study addressed the following research questions:

1. Do various country inequality aspects moderate the relationship between LCA and health?
2. Is the effect of LCA and country inequality on health, as well as the combination of those two, significant over two measurement time points, over three years?

## Method

### Sample and data

The analysis made use of data from two waves of SHARE, a biennial longitudinal survey of adults aged 50 and over, and their spouses of any age, in 27 European countries and Israel (31). Wave 7 of the survey (collected during 2017) included the SHARELIFE retrospective questionnaire, as well as background and health variables. In addition, eight new countries joined SHARE in Wave 7, encompassing full coverage of all continental EU Member States and allowing a comprehensive picture when using country-level analysis. Wave 8 (2019/2020, prior to the COVID outbreak) added another point of measurement to the main survey. To reduce selectivity due to mortality and morbidity, respondents older than 80 years at the time of their baseline interview were removed, as seniors older than 80 constitute a different population with differentiated patterns of health (32, 33).

The initial sample included 55,091 respondents aged 50–80, who had data in the retrospective questionnaire of Wave 7. From them were included only those who also participated in Wave 8: 29,117 (52.8% of the original sample). Another 328 participants (0.6% of the original sample) were removed due to missing information on some study variables. Therefore, the resultant sample numbered 28,789 persons from 25 countries: Austria, Belgium, Bulgaria, Croatia, Cyprus, Czechia, Denmark, Estonia, Finland, France, Germany, Greece, Hungary, Italy, Israel, Latvia, Lithuania, Luxembourg, Malta, Poland, Slovakia, Slovenia, Spain, Sweden and Switzerland.

### Variables

*Self-rated health* (34, 35) was examined in SHARE by means of a single question: “How would you say your health is these days? Would you say your health is excellent, very good, good, fair, poor?” This question is considered a robust measure of health status (36). The scale ranged from 1 to 5, with a higher score indicating a poorer assessment of health. Measurements from Wave 7 and Wave 8 were used in the current study as baseline and follow-up.

*Lifetime Cumulative Adversity (LCA)* was the first of the two independent variables of particular interest to the present study and was assessed by a list of potentially stressful life events (3). The LCA measure was built by Shrira (5) and has been used in studies based on SHARE (4, 8). It included self-reported events that met the criteria for traumatic or adjustment disorder events in DSM-5 (37). The original LCA was built from a Wave 3 questionnaire, and consisted of 15 difficult life events. In this study, the variable was assessed using the SHARELIFE questionnaire from SHARE Wave 7. Due to changes in the Wave 7 questionnaire, the present measurement included only 13 events; the two items that were removed were the birth of a stillborn baby and being raised by alcoholic parents/guardians. The remaining 13 events reflect four categories: early familial adversity, persecution, late familial adversity, and other adversities (Table 1). Physical health vulnerabilities were excluded to avoid confounding the health outcomes (8). The participants indicated whether such an event had ever occurred (1) or not (0). To reduce the impact of outliers, the number of adverse events experienced by participants was truncated at four, resulting in a scale ranging from 0 to 4. The LCA analysis included participants who experienced 0 (*n* = 16,205), 1 (*n* = 9,370), 2 (*n* = 2,470), 3 (*n* = 595), and 4 or more events (*n* = 149, including respondents reporting between 4 and 13 events). For more information, please refer to Table 1.

**Table 1:** Descriptive statistic for LCA—occurrence of adversities, separate, and total (*N* = 28,789).

	*N*	%
Early familial adversity		
Fostering with another family	323	1.1
Residing in a children’s home	434	1.5
Persecution		
Concentration camp	15	0.1
Discrimination	1,514	5.3
Dispossession of property due to persecution	2,097	7.3
Evacuation/relocation during war	518	1.8
Labor camp	43	0.2
War camp	21	0.1
Late familial adversity		
Deceased child	1,634	5.7
Deceased partner	3,932	13.7
Relationship breakdown/divorce	5,974	20.8
Other adversities		
Homelessness (1 month or more)	93	0.3
Prison	117	0.4
Total number of adversities – LCA		
0	16,205	56.3
1	9,370	32.6
2	2,470	8.6
3	595	2.1
4+	149	0.5

The second key independent variable—country—was examined in terms of three indicators: *Gini coefficient, democracy index,* and *welfare regimes*. The *Gini coefficient* is the most frequently used measure of income inequality; it ranges from 0 to 1, where 0 represents complete equality and 1 represents maximum inequality (the hypothetical condition of one person holding all income) (38). In the current study the values are represented as percentages from 0 to 100. A low Gini coefficient reflects greater equity in income distribution in a given country, whereas a high Gini coefficient reflects greater levels of inequality among earners. The Gini values for the baseline measurement point (2017) were taken from OECD (Organization for Economic Co-operation and Development) tables. The *democracy in*dex (39) is based on the ratings for 60 indicators grouped in five categories: electoral process and pluralism, civil liberties, functioning of the government, political participation, and political culture. Each category has a rating on a 0 to 10 scale. The overall index is the simple average of the five category indexes. A high democracy score reflects higher levels of democratic values in a country. *Welfare regimes* refers to Ferrera’s typology, expanded by Eikemo et al. (23, 40, 41). The European countries were classified into four types of welfare state regimes: Scandinavian (Northern Europe countries: Denmark, Finland, Sweden), Bismarckian (Western Europe countries: Austria, Belgium, Germany, France, Luxembourg), Southern (Cyprus, Greece, Italy, Israel, Portugal, Spain), and Eastern (Bulgaria, Croatia, Czechia, Estonia, Hungary, Latvia, Lithuania, Malta, Poland, Romania, Slovakia, Slovenia). For more information regarding the country distributions according to the three indicators, see Supplementary Table S1.

The analyses controlled for background sociodemographic variables, retrieved from SHARE Wave 7. These background variables included age, gender (male = 0, female = 1), living arrangement (no live-in partner = 0, live-in partner =1), education (recorded as one of seven levels according to the International Standard Classification of Educational Degrees—ISCED-97), and financial capacity (a 4-point scale, a higher score indicates fewer difficulties in making ends meet).

### Statistical analysis

First, descriptive statistics were calculated for all study variables (Continuous, or dummies for categorical variables), followed by Pearson correlations with SRH at baseline and follow-up. In addition, within each country, Pearson correlations were computed for the association between LCA and SRH, in order to evaluate whether the correlations varied among countries.

In the main analysis, Linear regressions using a multilevel approach were used to account for the nested nature of the data. That is, 28,789 individuals (Level 1) were nested within 25 countries (Level 2). A series of models were fitted. The baseline model, Model 0, included only the dependent variable SRH. Model A examined individual-level LCA as a predictor of SRH at follow-up, controlling for covariates (age, gender, partner, education, income). Thereafter, Model B was added to Model A by the Gini coefficient as a country-level predictor and cross-level interaction between LCA and Gini. Models C and D included identical analysis to Model B, but with democracy index and welfare regimes variables instead of Gini, respectively. The models were run twice, once without baseline measurement of SRH, and once with the baseline measurement in order to investigate cross-sectional versus change in SRH between the two waves. The relevant variables—LCA, Gini, and democracy—were centered to the grand mean for the analyses of the interactions. Graphs of the significant interactions were plotted with high and low levels of the country-level variables. All analyses were conducted using STATA 15.

## Results

Table 1 presents the frequency of occurrence for each of the 13 events of adversity by category. “Late familial” adversity events were the most frequently reported, whereas events in the “other adversities” category were the least frequent. The most frequent adverse event experienced by participants was relationship breakdown, with 20.8% reporting divorce or the end of a relationship during their lifetime. The least common life events were living in a concentration camp or war camp, with 0.1% of the participants. In the overall distribution of experiencing LCA, about 56% did not experience adversities at all, about one-third reported experiencing one adversity, and percentages declined consistently to approximately 3% experiencing three or more adversities. The mean LCA score was 0.58 (Table 2), indicating respondents experienced less than one adverse life event on average.

**Table 2:** Univariate and bivariate description of study variables (*N* = 28,789).

Characteristic	Mean	SD	Range	Self-rated health –baseline R	Self-rated health – follow-up R
Individual level					
Self-rated health – baseline	2.80	1.03	1–5		
Self-rated health – follow-up	2.79	1.01	1–5	0.607***	
Age	65.25	7.68	50–80	−0.200***	−0.220***
Gender (female)	0.57	0.49	0/1	−0.036***	−0.026***
Partner in HH	0.73	0.44	0/1	0.090***	0.071***
Education	3.11	1.35	0–6	0.149***	0.188***
Income	2.77	0.99	1–4	0.245***	0.258***
LCA	0.58	0.78	0–4	−0.122***	−0.097***
Country level					
Gini coefficient	29.69	4.04	23.20–40.20	−0.118***	−0.151***
Democracy level	7.88	0.82	6.49–9.39	0.123***	0.159***
Scandinavian regime	0.12	0.12	0/1	0.132***	0.166***
Bismarckian regime	0.25	0.25	0/1	0.048***	0.074***
Southern regime	0.13	0.13	0/1	0.084***	0.044***
Eastern regime	0.50	0.49	0/1	−0.182***	−0.200***

***p < 0.001.

Descriptive statistics and correlations of study variables are shown in Table 2. The mean age of the participants was 62, and there were more women (57%) in the sample than men. Slightly fewer than three-quarters of respondents had a live-in partner, and the mean education was slightly more than upper secondary education. With respect to financial capacity, the average sample member was able to make ends meet somewhat easily. Regarding SRH, respondents reported they were nearly in good health at baseline and follow-up, with a slightly better report at baseline. As for the country-level variables, the Gini coefficient stood at almost 30 in the sample, and the democracy index at 7.9. Half the sample were classified as Eastern regimes, a quarter as Bismarckian, and 12 and 13% as Scandinavian and Southern regimes, respectively. A more specific breakdown of the study variables by country is shown in Supplementary Table S1.

**Figure 1:**
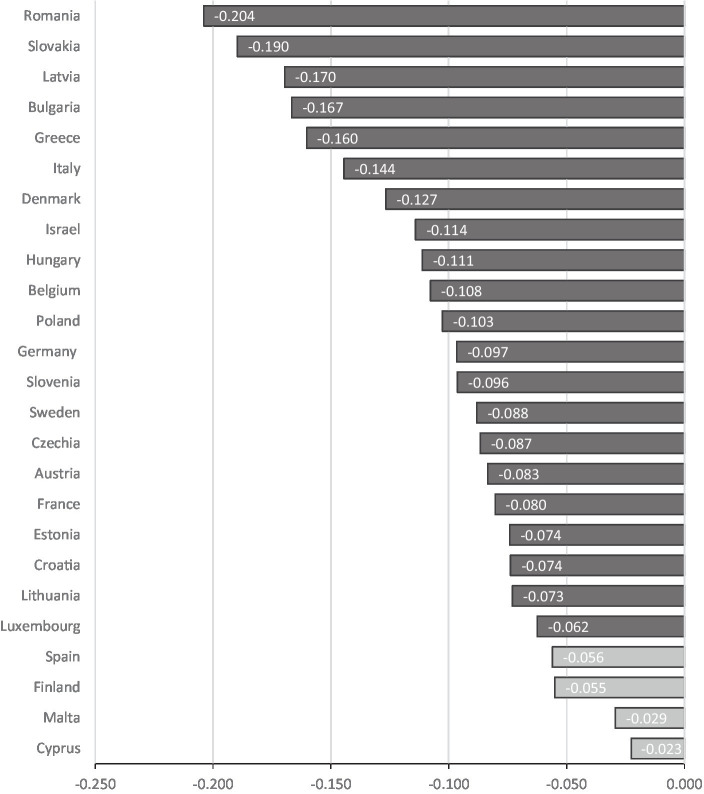
Pearson correlations between LCA and self-rated health at follow-up by country. Light gray countries have non-significant correlation between LCA and Self-rated health.

The bivariate correlations are also shown in Table 1. The analysis revealed that all the study variables were related to SRH measures at both measurement points. Older individuals and females had lower SRH than younger individuals and men. Partner status, education, and financial capacity were positively related to SRH. Higher levels of LCA were related to worse SRH. The Gini coefficient and Eastern regime were negatively correlated with SRH, while level of democracy and the three other regimes showed the opposite direction. The bivariate correlations of LCA with SRH for each country are presented in Figure 1. Some Eastern European countries (Romania, Slovakia, Latvia) were characterized in the higher Pearson correlation between LCA and health. Other Eastern European countries (Croatia and Lithuania) and one Western (Luxemburg) showed the lowest significant correlations. Spain, Finland, Malta, and Cyprus did not show significant correlations.

Four separate multilevel regression analyses were calculated to test the individual-level predictors and three country-level predictors (Gini, democracy, welfare regime) of SRH. as noted, these four regressions were calculated twice—one set for a single measurement of SRH to investigated cross-sectional effect, and the second set controlled for the baseline SRH to reflect the change in SRH between the two waves. The advantage of multilevel analysis is that it can take into account the dependency of observations among respondents from the same country.

The multilevel analyses for the change in SRH (controlled for baseline SRH) are shown in Table 3. As may be seen, the associations with the background and health variables were mostly consistent in all the regressions, in all the models. Thus, SRH at the second measurement point was positively correlated with baseline SRH, having higher education and higher income. Conversely, SRH was negatively correlated with age. No effects were found with reference to gender and partner status. Similar results were found in the series of regressions calculated without controlling for baseline SRH (Supplementary Table S2). Model A included only the individual level, adding LCA to backgrounds, while taking into account dependencies within countries. The model showed no significant associations between LCA and SRH. The same Model A for the SRH cross-sectional effect revealed different results: LCA was negatively associated with SRH. In Models B, C, and D, country-level variables were added separately, with interaction terms between those variables and LCA. Model B revealed a significant negative association between the Gini coefficient and SRH, but non-significant interaction of Gini and LCA. In Model C, the democracy index was positively correlated with SRH, and the interaction with LCA was negatively significant as well. Model D illustrated welfare regimes, with the reference category of the Scandinavian regime. Only the Eastern regime showed a significantly negative difference from the Scandinavian regime. As for the interactions, all three regimes—Bismarckian, Southern, and Eastern—were significantly different in relation to the Scandinavian regime. The same results were replicated in the other series of regressions, without controlling for baseline SRH.

**Table 3:** Multilevel regression models with individual and country variables predicting change in self-rated health between two points (*N* = 28,789).

	Self-rated health–follow-up				
	Model 0 – null	Model A – individual level	Model B – Gini	Model C – democracy	Model D – welfare regimes
	*b* (*SE _b_*)	*b* (*SE _b_*)	*b* (*SE _b_*)	*b* (*SE _b_*)	*b* (*SE _b_*)
Baseline					
Self-rated health		0.501*** (0.005)	0.501*** (0.005)	0.501*** (0.004)	0.501*** (0.005)
Backgrounds					
Age		−0.015*** (0.001)	−0.015*** (0.001)	−0.015*** (0.001)	−0.015*** (0.001)
Gender (female)		0.005 (0.010)	0.005 (0.010)	0.005 (0.009)	0.004 (0.009)
Partner in household		−0.022 (0.012)	−0.022 (0.012)	−0.022 (0.012)	−0.022 (0.012)
Education		0.065*** (0.004)	0.065*** (0.004)	0.064*** (0.004)	0.065*** (0.004)
Income		0.081*** (0.006)	0.081*** (0.006)	0.080*** (0.006)	0.080*** (0.006)
Independent					
LCA		−0.005 (0.007)	−0.004 (0.005)	−0.004 (0.005)	−0.056*** (0.015)
Country-level					
Gini coefficient			−0.051* (0.028)		
Gini * LCA			0.006 (0.005)		
Democracy level				0.066* (0.028)	
Democracy * LCA				−0.010* (0.005)	
Welfare regimes:ᵃ					
Bismarckian					−0.098 (0.085)
Southern					−0.095 (0.087)
Eastern					−0.254** (0.078)
Bismarckian * LCA					0.048** (0.017)
Southern * LCA					0.047* (0.023)
Eastern * LCA					0.066*** (0.016)
Constant	***2.813 (0.063)	1.949*** (0.058)	1.952*** (0.060)	1.956*** (0.060)	2.117*** (0.087)
Chi-squared	3077.52	***840.4	***726.4	704.2***	***562.6
VPC	0.094	0.036	0.032	0.029	0.023

*p < 0.05, **p < 0.01, ***p < 0.001; VPC – The variance partition coefficient. ᵃReference regime: Scandinavian.

In order to illustrate these results, graphs of the significant interactions are presented. The first graph in Figure 1 shows SRH by LCA according to the level of democracy (low or high). As may be seen, as LCA increases, SRH decreases. We also see, however, that regardless of LCA, high democracy is always related to better SRH than is low democracy. The effect of the interaction is apparent when comparing the respective ends of the slopes. On the left side of the graph, at the lowest LCA, the distances between the slopes of democracy are the widest. On the right side of the graph, at the highest LCA, the distances between the slopes of democracy are the narrowest. This shows that as LCA increases, the positive effect of democracy on SRH is reduced. Stated differently, democracy minimizes the risk of poor SRH among those with lower levels of LCA, but its effect is reduced as individuals have more LCA.

**Figure 2:**
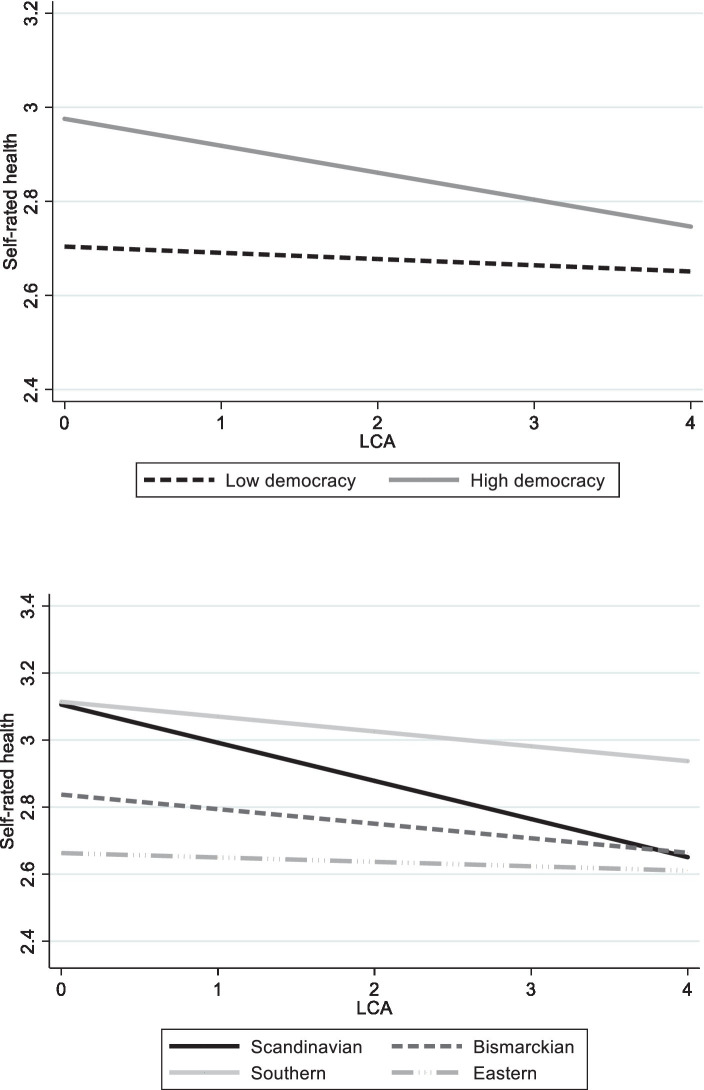
Self-rated health by LCA according to level of democracy and welfare regimes.

Figure 2 demonstrates the dynamic of LCA with welfare regimes. The Bismarckian, Southern, and Eastern regimes showed quite consistent effects: More LCA decreased SRH; although here, too, the gap between the slopes became somewhat narrower when LCA increased. As for the Scandinavian regime, the levels of LCA were crucial, and this slope decreased significantly compared to the other regimes. That is, an increase in LCA was dominant and had the highest relation to SRH deterioration in the Scandinavian regime.

## Discussion

The current inquiry considered 13 adverse life events and three country inequality indices vis-à-vis two points of SRH measure in old age. First of note is that LCA was found to be associated with SRH, net of the other study variables, only in a cross-sectional method but not in the two-points measurement time. These findings are consistent with a recent longitudinal study that indicated a significant effect of LCA on SRH at one point in time, but a relatively neglected effect over time (4). Authors suggested that stressful events fraught with traumatic reactions, may construct a complex idea of responses that does not always result in the same effect or outcome, depending on other factor. In line with this argument, the literature indicates many factors that can moderate the association between stress, adversity, and old-age health (7, 42–44). As such, the current study aimed to investigate the effect of LCA with country-level factors and did reveal that the long-term associations between LCA and health are not always significant but may become pronounced only with the presence of other factors.

Country-level inequality indices were also significant predictors of SRH, both cross-sectional and with two points in time. Moreover, they worked primarily in the directions that have been variously reported in the literature (20, 23, 45). That is, less inequality in a country (lower Gini coefficient, higher democracy score, living at a Scandinavian welfare regime) coincides with greater SRH. The present finding, resulting from a large representative sample, thus reconfirms that inequalities at the macro level may adversely be related with population health, and that inequality is related to a person’s perceived health in later life, as noted in the literature (17, 18).

The first research question to be considered in the analysis was whether country inequality indices intervene in the association between LCA and SRH. The results of the present analysis show that they partly do. Generally, in the bivariate analysis, it was observed that in most counties, LCA was negatively associated with SRH, but at different levels of significance. The multivariate analysis revealed that democracies and welfare regimes modify the ill effects of LCA on health, but this effect is reduced as the LCA level increases. To state it differently, low inequality may positively correlate with health and alleviate to some extent the consequences of a person’s adverse life history; however, high levels of adverse life events suppress that positive effect. More specifically, democratic and welfare regimes were found to interact with the effects of LCA on SRH, but the Gini coefficient did not. In accordance with cumulative inequality theory (2), individual levels (LCA) together with social levels (democracy levels and welfare regimes) were found in this inquiry to shape the trajectories of inequality in life. A high level of democracy is related to high levels of SRH across all levels of LCA, and Scandinavian and Southern regimes had the highest levels of SRH across the levels of LCA, followed by Bismarckian, while the least was the Eastern regime. Nevertheless, the relative advantage of democracy and some of the welfare regimes was reduced as LCA increased. This can be explained due to cumulative adversities possibly being offset with better societal features and low inequality: Although Democratic political values tend to have lower levels of inequality (46), and Scandinavian countries have social benefits and services that were found to buffer the structural pressures on health inequalities (47, 48), it seems that country can compensate for individual adversity only to a limited degree. In line with cumulative inequality theory (2), both individual risk and social factors are crucial to shape the accumulation of inequity in health.

The research yielded several additional noteworthy findings concerning the interaction between country-level inequality and the individual LCA. The Southern European regime was the highest to correlate with SRH, along with the Scandinavian regime. This association remains relatively high also at the higher levels of LCA, as opposed to the Scandinavian regime. This finding may indicate that not only that lower rates of inequality, reduces cumulative stress and hence positively affects the health state, but also social environment and relationships. The Southern regime includes countries whose institutions are less supportive of equal distribution of resources than the Scandinavian or Bismarckian (41). However, the Southern countries are characterized by a high level of social cohesion and a sense of community, with studies reporting a strong “familistic” cultural tradition in Mediterranean countries and high levels of social support (49, 50). These social dimensions have previously been found to represent a protective factor both in reducing the vulnerability of older people and in helping individuals cope with stress and adversity (51, 52). This finding supplements the fact that the Gini coefficient does not interact with LCA regarding health outcome; that is, it positively related to better SRH regardless of a person’s individual history of distress. It is possible to conclude that a variety of social dimensions, other than economic inequality or welfare policies, can also contribute to health outcomes in old age along with LCA.

On the other hand, as mentioned, the Scandinavian regime, which is characterized by the most generous and universal welfare policies (40), was associated with better health status at low levels of LCA, as expected from the view of inequality theory, but high levels of adverse life events “outweighed” the advantage that these countries provide. This result can be attributed to the northern “paradox” found in some studies, indicating that although Scandinavian countries have policies directed to reduce inequality (40, 53), they do not consistently exhibit the lowest health inequities (15, 54). Various factors have been suggested to explain this puzzle or paradox, including differences in inequities in health-related behaviors across welfare regimes (55). One study suggests that this welfare state contributes to widening health inequities by increasing the availability of certain health-damaging goods, such as alcohol and tobacco (56). The use of those materials was also found to represent an unsuccessful coping strategy for stress and adversity (57). Further study should deeply investigate this assumption, to understand the different patterns of the correlation between LCA and SRH found in the Scandinavian regime in the present study.

The second research question queried whether the effect of LCA and country inequality indices on SRH, as well as the combination of those two, would remain significant over two measurement time-points over three years. The results of the present study demonstrate that moderation effect of LCA and country indices remained the same in the two points of measurement. These findings correspond with the assumptions of cumulative inequality theory, which suggests life course trajectories are influenced by accumulated inequalities and the loss of resources, which in turn may lead to an accelerated aging process over time (2). The literature argues that it is important to examine the consequences of accumulating traumas throughout life (7), although only a few studies have examined these associations over a long period in late life f. The current findings extend the application of this theory about the consequences of adversity over a lifetime by examining LCA in conjunction with country inequality and its effects on the health changes in old age. However, this investigation applying long-term analysis is only preliminary, since it uses only two points in time.

A few limitations of the present study should be noted. First, LCA is reliant on retrospective reports, which may in some cases be subject to recall bias. This inaccuracy of recall may derive from simple recall failure but may also stem from other causes, such as applying a present lens to “color” a past situation, or depression, which may increase the tendency to remember and report difficult life events (58). Another limitation lies in the reliance on the retrospective events in Wave 7 rather than Wave 3, as was investigated in previous studies (4, 5). As noted in the method section, some events were missing in the Wave 7 version. Thus, the results of this study are valid only for the list of life events examined.

As a limitation of the moderator variables, it should be noted that these variables – GINI coefficient, welfare regimes, and democratic levels – have certain limitations that must be acknowledged when analyzing differences between countries. The GINI coefficient may fails to capture nuances within societal distributions and may overlook factors such as wealth disparities or access to essential services (59). Similarly, categorizing countries into distinct welfare regimes may oversimplify complex social welfare systems, potentially neglecting variations within each regime and failing to account for evolving social policies. Lastly, assessing democratic levels through scales may overlook subtle authoritarian tendencies or cultural, historical, and contextual factors that influence the functioning of democratic institutions (20). It is important to interpret these scales carefully and understand their contextual nature.

In conclusion, the present study’s findings support the cumulative inequality theory by showing that life course trajectories may be influenced by an individual’s accumulative risk exposure to stress and inequities at the societal level. The results demonstrated that LCA was negatively associated with the change between two point-in-time trajectories of SRH, with dependence on the country-level inequality characteristics. It revealed that inequality at the country level, indeed intervenes in the LCA–health nexus. These results have practical implications. Given the rapidly aging population in many countries, understanding the country-level factors associated with health deterioration is extremely important, especially for policymakers. Because it is easier to adopt policies to modify a country’s inequity than it is to modify an individual’s lifetime cumulative stressful events, this observation provides constructive and important guidance for decreasing the harmful effects of lifetime adversity in old age.

## Data availability statement

Publicly available datasets were analyzed in this study. This data can be found at: https://share-eric.eu/data/data-access.

## Ethics statement

The studies involving humans were approved by the Ethics Council of the Max Planck Society. The studies were conducted in accordance with the local legislation and institutional requirements. The participants provided their written informed consent to participate in this study.

## Author contributions

ML: Writing – original draft, Writing – review & editing.

## References

[1] Rechel B Grundy E Robine JM Cylus J MacKenbach JP Knai C. Ageing in the European Union. Lancet. (2013) 381:1312–22. doi: 10.1016/S0140-6736(12)62087-X 23541057

[2] Ferraro KF Shippee TP. Aging and cumulative inequality: how does inequality get under the skin? Gerontologist. (2009) 49:333–43. doi: 10.1093/geront/gnp034, PMID: 19377044 PMC2721665

[3] Turner RJ Lloyd DA. Lifetime traumas and mental health: the significance of cumulative adversity. J Health Soc Behav. (1995) 36:360–76. doi: 10.2307/2137325, PMID: 8719054

[4] Levinsky M Schiff M. Lifetime cumulative adversity and physical health deterioration in old age: evidence from a fourteen-year longitudinal study. Soc Sci Med. (2021) 289:114407. doi: 10.1016/J.SOCSCIMED.2021.114407, PMID: 34555682

[5] Shrira A. The effect of lifetime cumulative adversity on change and chronicity in depressive symptoms and quality of life in older adults. Int Psychogeriatr. (2012) 24:1988–97. doi: 10.1017/S1041610212001123, PMID: 22874666

[6] Kinan G Shrira A Shmotkin D. The association between cumulative adversity and mental health: considering dose and primary focus of adversity. Qual Life Res. (2012) 21:1149–58. doi: 10.1007/s11136-011-0035-0, PMID: 21983715

[7] Seery MD Holman EA Silver RC. Whatever does not kill us: cumulative lifetime adversity, vulnerability, and resilience. J Pers Soc Psychol. (2010) 99:1025–41. doi: 10.1037/a0021344 20939649

[8] Shrira A Litwin H. The effect of lifetime cumulative adversity and depressive symptoms on functional status. J Gerontol Series B Psychol Sci Soc Sci. (2014) 69:953–65. doi: 10.1093/geronb/gbu056, PMID: 24898028 PMC4296139

[9] Shrira A. Greater age-related decline in markers of physical, mental and cognitive health among Israeli older adults exposed to lifetime cumulative adversity. Aging Ment Health. (2014) 18:610–8. doi: 10.1080/13607863.2013.860951, PMID: 24328416 PMC4021036

[10] Di Ciaula A Portincasa P. The environment as a determinant of successful aging or frailty. Mech Ageing Dev. (2020) 188:111244. doi: 10.1016/J.MAD.2020.111244 32335099

[11] Filho ADPC Kawachi I Wang YP Viana MC Andrade LHSG. Does income inequality get under the skin? A multilevel analysis of depression, anxiety and mental disorders in São Paulo. Brazil J Epidemiol Community Health. (2013) 67:966–72. doi: 10.1136/JECH-2013-202626, PMID: 23908459

[12] Schmidt CW. Environmental factors in successful aging: the potential impact of air pollution. Environ Health Perspect. (2019) 127:102001. doi: 10.1289/EHP4579, PMID: 31573833 PMC6910773

[13] Garin N Koyanagi A Chatterji S Tyrovolas S Olaya B Leonardi M. Global multimorbidity patterns: a cross-sectional, population-based, multi-country study. J Gerontol Series A. (2016) 71:205–14. doi: 10.1093/GERONA/GLV128, PMID: 26419978 PMC5864156

[14] Mackenbach JP Valverde JR Artnik B Bopp M Brønnum-Hansen H Deboosere P. Trends in health inequalities in 27 European countries. Proc Natl Acad Sci USA. (2018) 115:6440–5. doi: 10.1073/pnas.1800028115, PMID: 29866829 PMC6016814

[15] Eikemo TA Bambra C Joyce K Dahl E. Welfare state regimes and income-related health inequalities: a comparison of 23 European countries. Eur J Pub Health. (2008) 18:593–9. doi: 10.1093/eurpub/ckn092, PMID: 18927186

[16] Olsen KM Dahl SÅ. Health differences between European countries. Soc Sci Med. (2007) 64:1665–78. doi: 10.1016/J.SOCSCIMED.2006.11.031 17250940

[17] Bjørnskov C. Determinants of generalized trust: a cross-country comparison. Public Choice. (2007) 130:1–21. doi: 10.1007/s11127-006-9069-1

[18] Freitag M Bühlmann M. Crafting trust: the role of political institutions in a comparative perspective. Comp Pol Stud. (2011) 42:1537–66. doi: 10.1177/0010414009332151

[19] Kawachi I Kennedy BP Glass R. Social capital and self-rated health: a contextual analysis. Am J Public Health. (1999) 89:1187–93. doi: 10.2105/AJPH.89.8.1187, PMID: 10432904 PMC1508687

[20] Gugushvili A Reeves A. How democracy alters our view of inequality—and what it means for our health. Soc Sci Med. (2021) 283:114190. doi: 10.1016/J.SOCSCIMED.2021.114190, PMID: 34242889

[21] Madero-Cabib I Corna L Baumann I. Aging in different welfare contexts: a comparative perspective on later-life employment and health. J Gerontol Series B Psychol Sci Soc Sci. (2020) 75:1515–26. doi: 10.1093/geronb/gbz037, PMID: 30888038 PMC7424286

[22] Ploubidis GB Dale C Grundy E. Later life health in Europe: how important are country level influences? Eur J Ageing. (2012) 9:5–13. doi: 10.1007/s10433-011-0215-3, PMID: 28804403 PMC5547417

[23] Eikemo TA Bambra C Judge K Ringdal K. Welfare state regimes and differences in self-perceived health in Europe: a multilevel analysis. Soc Sci Med. (2008) 66:2281–95. doi: 10.1016/j.socscimed.2008.01.022, PMID: 18314241

[24] Sieber S Cheval B Orsholits D Van Der Linden BW Guessous I Gabriel R. Welfare regimes modify the association of disadvantaged adult-life socioeconomic circumstances with self-rated health in old age. Int J Epidemiol. (2019) 48:1352–66. doi: 10.1093/ije/dyy283, PMID: 30608584 PMC6934032

[25] Brandt M Deindl C Hank K. Tracing the origins of successful aging: the role of childhood conditions and social inequality in explaining later life health. Soc Sci Med. (2012) 74:1418–25. doi: 10.1016/J.SOCSCIMED.2012.01.004, PMID: 22398143

[26] van der Linden BWA Sieber S Cheval B Orsholits D Guessous I Gabriel R. Life-course circumstances and frailty in old age within different European welfare regimes: a longitudinal study with SHARE. J Gerontol. (2020) 75:1326–35. doi: 10.1093/geronb/gbz140 PMC7265805 31665484

[27] Darin-Mattsson A Fors S Kåreholt I. Different indicators of socioeconomic status and their relative importance as determinants of health in old age. Int J Equity Health. (2017) 16:173. doi: 10.1186/s12939-017-0670-3, PMID: 28950875 PMC5615765

[28] Makaroun LK Brown RT Diaz-Ramirez LG Ahalt C Boscardin WJ Lang-Brown S. Wealth-associated disparities in death and disability in the United States and England. JAMA Intern Med. (2017) 177:1745–53. doi: 10.1001/jamainternmed.2017.3903, PMID: 29059279 PMC5820733

[29] Ohrnberger J Fichera E Sutton M. The relationship between physical and mental health: a mediation analysis. Soc Sci Med. (2017) 195:42–9. doi: 10.1016/j.socscimed.2017.11.008 29132081

[30] Zimmer Z Hanson HA Smith KR. Childhood socioeconomic status, adult socioeconomic status, and old-age health trajectories: connecting early, middle, and late life. Demogr Res. (2016) 34:285–320. doi: 10.4054/DemRes.2016.34.10

[31] Börsch-Supan A Brandt M Hunkler C Kneip T Korbmacher J Malter F. Data resource profile: the survey of health, ageing and retirement in europe (share). Int J Epidemiol. (2013) 42:992–1001. doi: 10.1093/ije/dyt088, PMID: 23778574 PMC3780997

[32] Crimmins EM. Trends in the health of the elderly. Annu Rev Public Health. (2004) 25:79–98. doi: 10.1146/ANNUREV.PUBLHEALTH.25.102802.124401 15015913

[33] Wauters M Elseviers M Vaes B Degryse J Vander Stichele R Christiaens T. Mortality, hospitalisation, institutionalisation in community-dwelling oldest old: the impact of medication. Arch Gerontol Geriatr. (2016) 65:9–16. doi: 10.1016/j.archger.2016.02.009, PMID: 26913791

[34] Krause NM Jay GM. What do global self-rated health items measure? Med Care. (1994) 32:930–42. doi: 10.1097/00005650-199409000-00004 8090045

[35] Markides KS Martin HW. Predicting self-related health among the aged. Res Aging. (1979) 1:97–112. doi: 10.1177/016402757911006

[36] Pietilä O Laaksonen M Rahkonen O Lahelma E Van Baal PHM. Self-rated health as a predictor of disability retirement-the contribution of ill-health and working conditions. J PLoS Org. (2011) 6:5004. doi: 10.1371/journal.pone.0025004, PMID: 21949830 PMC3176797

[37] Lyter SC Lyter LL. Diagnostic and statistical manual of mental disorders. Int J Interdiscipl Soc Sci Ann Rev. (2016) 6:53–64. doi: 10.18848/1833-1882/cgp/v06i06/52093

[38] Subramanian SV Kawachi I. Income inequality and health: what have we learned so far? Epidemiol Rev. (2004) 26:78–91. doi: 10.1093/epirev/mxh003, PMID: 15234949

[39] Democracy Index. (2017). Free speech under attack. The Economist Intelligence Unit.

[40] Esping-Anderson G. The three worlds of welfare capitalism. Princeton University Press. (1990) 70:532. doi: 10.2307/2580262

[41] Ferrera M. The ‘southern model’ of welfare in social Europe. J Eur Soc Policy. (1996) 6:17–37. doi: 10.1177/095892879600600102

[42] Chen S Westman M Hobfoll SE. The commerce and crossover of resources: resource conservation in the service of resilience. Stress Health. (2015) 31:95–105. doi: 10.1002/smi.2574, PMID: 25873421 PMC4564014

[43] Stein JY Bachem R Lahav Y Solomon Z. The aging of heroes: posttraumatic stress, resilience and growth among aging decorated veterans. J Posit Psychol. (2020) 16:390–7. doi: 10.1080/17439760.2020.1725606

[44] Taylor MG Ureña S Carr DC Min S. Early-life military exposures and functional impairment trajectories among older male veterans: the buffering effect of psychological resilience. J Gerontol. (2019) 74:832–41. doi: 10.1093/GERONB/GBY029, PMID: 29788363

[45] Inaba Y Wada Y Ichida Y Nishikawa M. Which part of community social capital is related to life satisfaction and self-rated health? A multilevel analysis based on a nationwide mail survey in Japan. Soc Sci Med. (2015) 142:169–82. doi: 10.1016/J.SOCSCIMED.2015.08.007, PMID: 26310593

[46] Iveniuk J Laumann EO Waite LJ McClintock MK Tiedt A. Personality measures in the National Social Life, health, and aging project. J Gerontol Ser B Psychol Sci Soc Sci. (2014) 69:S117–24. doi: 10.1093/geronb/gbu073, PMID: 25360012 PMC4303096

[47] McCartney G Hearty W Arnot J Popham F Cumbers A McMaster R. Impact of political economy on population health: a systematic review of reviews. Am J Public Health. (2019) 109:E1–E12. doi: 10.2105/AJPH.2019.305001, PMID: 31067117 PMC6507992

[48] Navarro V Shi L. The political context of social inequalities and health. Int J Health Serv. (2001) 31:1–21. doi: 10.2190/1GY8-V5QN-A1TA-A9KJ 11271636

[49] Kalmijn M Saraceno C. A comparative perspective on intergenerational support: responsiveness to parental needs in individualistic and familialistic countries. Eur Soc. (2008) 10:479–508. doi: 10.1080/14616690701744364

[50] Litwin H. Social networks and well-being: a comparison of older people in Mediterranean and non-Mediterranean countries. J Gerontol Series B. (2010) 65B:599–608. doi: 10.1093/GERONB/GBP104, PMID: 20008485 PMC2920940

[51] DeLongis A O’Brien T. An interpersonal framework for stress and coping: an application to the families of Alzheimer’s patients. Stress Coping Later Life Fam. (2018):221–39. doi: 10.4324/9781315803074-13

[52] Melchiorre MG Chiatti C Lamura G Torres-Gonzales F Stankunas M Lindert J. Social support, socio-economic status, health and abuse among older people in seven European countries. PLoS One. (2013) 8:e54856. doi: 10.1371/journal.pone.0054856, PMID: 23382989 PMC3559777

[53] Arts W Gelissen J. Three worlds of welfare capitalism or more? A state-of-the-art report. J Eur Soc Policy. (2002) 12:137–58. doi: 10.1177/0952872002012002114

[54] Guarnizo-Herreño CC Watt RG Garzón-Orjuela N Tsakos G. Explaining oral health inequalities in European welfare state regimes: the role of health behaviours. Community Dent Oral Epidemiol. (2019) 47:40–8. doi: 10.1111/cdoe.12420, PMID: 30211446

[55] Bambra C. Health inequalities and welfare state regimes: theoretical insights on a public health ‘puzzle’. J Epidemiol Community Health. (2011) 65:740–5. doi: 10.1136/jech.2011.136333, PMID: 21690243

[56] Pega F Blakely T Carter K Sjöberg O. The explanation of a paradox? A commentary on Mackenbach with perspectives from research on financial credits and risk factor trends. Soc Sci Med. (2012) 75:770–3. doi: 10.1016/j.socscimed.2012.03.052, PMID: 22682368

[57] Corbin WR Farmer NM Nolen-Hoekesma S. Relations among stress, coping strategies, coping motives, alcohol consumption and related problems: a mediated moderation model. Addict Behav. (2013) 38:1912–9. doi: 10.1016/j.addbeh.2012.12.005, PMID: 23380486

[58] Mazzonna F Havari E. Can we trust older People’s statements on their childhood circumstances? Evidence from SHARELIFE. SSRN Electron J. (2011). doi: 10.2139/ssrn.2004299

[59] Manero A. The limitations of negative incomes in the Gini coefficient decomposition by source. Appl Econ Lett. (2017) 24:977–81. doi: 10.1080/13504851.2016.1245828

