# Supplementary material for: Can countries shape the association between cumulative adversity and old-age health?
Source: Front Public Health. 2024 May 15;12:1364868. doi: 10.3389/fpubh.2024.1364868 (PMC11133626; doi:10.3389/fpubh.2024.1364868)
Supplement: Supplementary file 1 [file Table_1.docx]

| **Supplementary Table 1.** Distribution of Country-Level Variables – Welfare Regimes, Gini Coefficient and Democracy Index | | | |
| --- | --- | --- | --- |
| **Welfare regime** | **Country** | **GINI** | **Democracy** |
| Scandinavian | Denmark | 27.6 | 9.22 |
|  | Finland | 25.3 | 9.25 |
|  | Sweden | 28.0 | 9.39 |
| Bismarckian | Austria | 27.9 | 8.29 |
|  | Belgium | 26.1 | 7.64 |
|  | Germany | 29.1 | 8.68 |
|  | France | 28.8 | 8.12 |
|  | Luxembourg | 29.2 | 8.81 |
| Southern | Cyprus | 30.8 | 7.59 |
|  | Greece | 33.4 | 7.43 |
|  | Italy | 32.7 | 7.52 |
|  | Israel | 35.1 | 7.86 |
|  | Portugal | 33.5 | 8.03 |
|  | Spain | 34.1 | 8.29 |
| Eastern | Bulgaria | 40.2 | 7.03 |
|  | Croatia | 29.9 | 6.57 |
|  | Czechia | 24.5 | 7.69 |
|  | Estonia | 31.6 | 7.90 |
|  | Hungary | 28.1 | 6.63 |
|  | Latvia | 34.5 | 7.49 |
|  | Lithuania | 37.6 | 7.50 |
|  | Malta | 28.2 | 7.95 |
|  | Poland | 29.2 | 6.62 |
|  | Romania | 33.1 | 6.49 |
|  | Slovakia | 23.2 | 7.17 |
|  | Slovenia | 23.7 | 7.50 |

| **Supplementary Table 2.**  Multilevel Regression Models with Individual and Country Variables Predicting Self-Rated (N = 28,789) | | | | | | | | |
| --- | --- | --- | --- | --- | --- | --- | --- | --- |
|  | **Self-rated health–**  **Follow-up** | | | | | | | |
|  | **Model A –**  **Individual level** | | **Model B -**  **GINI** | | **Model C –**  **Democracy** | | **Model D –**  **Welfare regimes** | |
|  | ***b*** | **(*SE* *_b_*)** | ***b*** | **(*SE* *_b_*)** | ***b*** | **(*SE* *_b_* )** | ***b*** | **(*SE* *_b_* )** |
|  |  |  |  |  |  |  |  |  |
| **Backgrounds** |  |  |  |  |  |  |  |  |
| Age | -0.026*** | (0.000) | -0.027*** | (0.001) | -0.027*** | (0.001) | -0.027*** | (0.001) |
| Gender (female) | -0.003 | (0.011) | -0.003 | (0.011) | -0.004 | (0.011) | -0.004 | (0.011) |
| Partner in household | -0.023 | (0.014) | -0.022 | (0.014) | -0.023 | (0.014) | -0.023 | (0.014) |
| Education | 0.110*** | (0.005) | 0.110*** | (0.006) | 0.110*** | (0.005) | 0.109*** | (0.005) |
| Income | 0.186*** | (0.006) | 0.186*** | (0.006) | 0.185*** | (0.006) | 0.185*** | (0.006) |
| **Independent** |  |  |  |  |  |  |  |  |
| LCA | -0.034*** | (0.008) | -0.026*** | (0.006) | -0.026*** | (0.006) | -0.087*** | (0.017) |
| **Country-level** |  |  |  |  |  |  |  |  |
| Gini coefficient |  |  | -0.064* | (0.053) |  |  |  |  |
| Gini * LCA |  |  | 0.007 | (0.006) |  |  |  |  |
| Democracy level |  |  |  |  | 0.091* | (0.052) |  |  |
| Democracy * LCA |  |  |  |  | -0.010* | (0.006) |  |  |
| Welfare regimesᵃ |  |  |  |  |  |  |  |  |
| Bismarckian |  |  |  |  |  |  | -0.163 | (0.164) |
| Southern |  |  |  |  |  |  | 0.001 | (0.169) |
| Eastern |  |  |  |  |  |  | -0.382* | (0.149) |
| Bismarckian * LCA |  |  |  |  |  |  | 0.057** | (0.020) |
| Southern * LCA |  |  |  |  |  |  | 0.055* | (0.024) |
| Eastern * LCA |  |  |  |  |  |  | 0.078*** | (0.019) |
|  |  |  |  |  |  |  |  |  |
| Constant | 3.740*** | (0.078) | 1.938*** | (0.060) | 3.732*** | (0.076) | 3.947*** | (0.145) |
| R-squared | 2463.9*** |  | 2287.6*** |  | 2243.3*** |  | 1707.7*** |  |

Notes: * p<0.05, ** p<0.01, *** p<0.001

ᵃ Reference regime: Scandinavian
